# Isolation and Characterization of Novel Lytic Phages Infecting Multidrug-Resistant Escherichia coli

**DOI:** 10.1128/spectrum.01678-21

**Published:** 2022-02-16

**Authors:** Javiera Vera-Mansilla, Patricio Sánchez, Cecilia A. Silva-Valenzuela, Roberto C. Molina-Quiroz

**Affiliations:** a Centro de Estudios Científicos, Valdivia, Chile; Institut Pasteur

**Keywords:** *Escherichia coli*, antibiotic resistance, multidrug-resistant clinical isolates, bacteriophages, urinary tract infections, antibiotic-resistant pathogens, LPS, phage-host interactions, phage predation, lipopolysaccharide

## Abstract

Urinary tract infections (UTIs) are the second most frequent bacterial infections worldwide, with Escherichia coli being the main causative agent. The increase of antibiotic-resistance determinants among isolates from clinical samples, including UTIs, makes the development of novel therapeutic strategies a necessity. In this context, the use of bacteriophages as a therapeutic alternative has been proposed, due to their ability to efficiently kill bacteria. In this work, we isolated and characterized three novel bacteriophages, microbes laboratory phage 1 (MLP1), MLP2, and MLP3, belonging to the *Chaseviridae*, *Myoviridae*, and *Podoviridae* families, respectively. These phages efficiently infect and kill laboratory reference strains and multidrug-resistant clinical E. coli isolates from patients with diagnosed UTIs. Interestingly, these phages are also able to infect intestinal pathogenic Escherichia coli strains, such as enteroaggregative E. coli and diffusely adherent E. coli. Our data show that the MLP phages recognize different regions of the lipopolysaccharide (LPS) molecule, an important virulence factor in bacteria that is also highly variable among different E. coli strains. Altogether, our results suggest that these phages may represent an interesting alternative for the treatment of antibiotic-resistant E. coli.

**IMPORTANCE** Urinary tract infections affect approximately 150 million people annually. The current antibiotic resistance crisis demands the development of novel therapeutic alternatives. Our results show that three novel phages, MLP1, MLP2, and MLP3 are able to infect both laboratory and multidrug-resistant clinical isolates of Escherichia coli. Since these phages (i) efficiently kill antibiotic-resistant clinical isolates of uropathogenic Escherichia coli (UPEC), (ii) recognize different portions of the LPS molecule, and (iii) are able to efficiently infect intestinal pathogenic Escherichia coli hosts, we believe that these novel phages are good candidates to be used as a therapeutic alternative to treat antibiotic-resistant E. coli strains generating urinary tract and/or intestinal infections.

## INTRODUCTION

Urinary tract infections (UTIs) account for millions of clinical cases worldwide per year, affecting mainly women and the elderly. Approximately 50% of women will have a UTI in their lifetime, and 20 to 30% will experience relapsing infections (1, 2). The severity of these infections can range from uncomplicated to complicated UTIs. Uncomplicated or acute clinical outcome (cystitis) UTIs are prevalent in healthy individuals. However, in patients with urinary obstruction, retention caused by pregnancy, renal failure, or indwelling catheters, a complicated UTI may cause kidney damage and, in the most severe cases, sepsis and death.

Uropathogenic Escherichia coli (UPEC) is the main etiological agent, accounting for 75% of uncomplicated and 65% of complicated UTI cases (3). It has been well established that this pathogen is harmless when residing in the human intestine. However, when it reaches the urinary tract, it adapts its metabolism, generating UTIs (4).

Due to their high prevalence and the increased antibiotic resistance (AR) reported in clinical isolates of UPEC, UTIs represent a serious public health issue worldwide (5, 6). Antimicrobial resistance has risen rapidly in recent years, generating an even more complex scenario where multidrug-resistant (MDR) UPEC strains are unresponsive to the currently available antibacterial therapies (7, 8). During the last 30 years, the discovery and approval of antibiotics has decreased by 90%. For this reason, most pharmaceutical companies have abandoned research and development in antibiotics (9). Antibiotic-resistant diseases already cause approximately 700,000 deaths a year globally, and the World Health Organization (WHO) has estimated that this number might increase to 10 million deaths by 2050, generating more deaths than cancer in the near future (10). Therefore, WHO has encouraged researchers to develop novel antibacterial strategies to treat a list of priority AR pathogens (11).

A promising alternative to treat infections caused by AR pathogens is the use of bacteriophages (phages). Phages are lytic viruses of bacteria present in diverse environments, such as soil, wastewater, and aquatic environments, among others (12). The use of phages as a therapeutic alternative is very promising due to their high specificity. These organisms exclusively infect their host and do not impact host microbiota, unlike broad-spectrum antibiotics, which can cause intestinal microbial dysbiosis (13). Indeed, the use of bacteriophages in clinical settings as treatment for UTIs has been evaluated with promising results (14–16).

Aiming to explore a complimentary or alternative strategy for the treatment of UTIs caused by MDR clinical UPEC isolates currently infecting people in our geographical location, we isolated and characterized three novel phages, named MLP1, MLP2, and MLP3. The phages were enriched using the UPEC reference strain CFT073 as the host, and we determined that they effectively infect and kill both laboratory strains and MDR clinical isolates of UPEC and intestinal pathogenic E. coli (InPEC) strains (hereinafter, both will be referred to as intestinal pathogenic E. coli, since UPEC resides in the human intestine, as mentioned above). Our work raises the potential of these phages to be used against current MDR strains associated with UTIs.

## RESULTS

### Phage isolation and genomic characterization.

**FIG 1:**
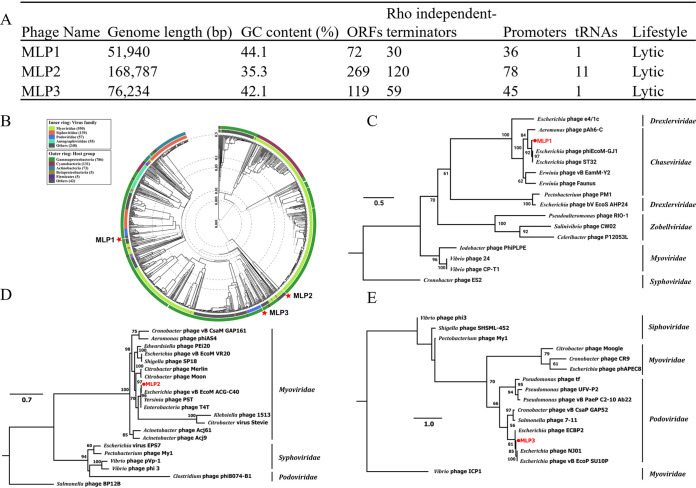
Genomic and phylogenetic characterization of novel phages. (A) Characterization of MLP phage genomes. (B) Proteome-based phylogenetic tree of phages constructed by comparison of 311 related taxa using VipTree. Inner and outer rings indicate phage and host taxa at the family level, respectively. (C to E) Phylogenetic analysis of MLP1 (C), MLP2 (D), and MLP3 (E). Bootstrap support (>60) is shown for each node.

Water samples collected from the Valdivia River (Chile) were used to enrich potential phages against UTI-associated E. coli by using the reference UPEC CFT073 strain as a host. Single plaques with different morphologies were picked and used to prepare bacterial lysates with an enriched phage titer (17) and to purify genomic DNA. Whole-genome sequencing (WGS) of phage genomes showed that all of the phages had a double-stranded-DNA genome and that they varied in genome size, GC content, and number of predicted open reading frames (ORFs), among other features (Fig. 1A). Interestingly, MLP2 exhibited a larger amount of encoded tRNAs than MLP1 and MLP3 (Fig. 1A). These elements correspond to codons used with a higher frequency to translate phage proteins and have been proposed to correlate with an improved virulence and fitness (18). Using 2 different bioinformatic tools (19, 20) we determined that MLP1, MLP2, and MLP3 had a lytic lifestyle (Fig. 1A).

Using the VipTree tool, we generated a proteome-based phylogenetic tree for taxonomic classification (21). This analysis showed that even when the three MLP phages displayed similarities to other phages infecting members of *Gammaproteobacteria*, they were not phylogenetically related to each other (Fig. 1B). Since (i) MLP1 was classified as “others” (Fig. 1B), (ii) phage taxonomy has changed during recent years and new families of myoviruses have been ratified (22), and (iii) we aimed to validate and/or improve the taxonomic classification of these phages, we carried out a phylogenetic analysis based on the amino acid sequence of the terminase large subunit (TerL). TerL is frequently used as a phylogenetic marker due to its high level of sequence conservation and its ubiquity among phages (23). After conducting these additional analyses, our results showed that MLP1 belongs to the *Chaseviridae* (Fig. 1C), MLP2 to the *Myoviridae* (Fig. 1D), and MLP3 to the *Podoviridae* family (Fig. 1E).

To identify exclusive traits of MLP phages, we used BLASTn (24) and chose the closest genome by percent identity (>90%). Alignments of MLP genomes with the most similar phage genomes in the RefSeq non-redundant proteins NCBI database were conducted using the ACT tool, starting the alignment in the *terL* gene (Fig. S1 in the supplemental material) (25). MLP1 and MLP3 showed different genomic arrangements than their most similar relatives from the NCBI database (Fig. S1). However, the alignment with the closest relative to MLP2 from the NCBI database showed high homology (98% in 96% of its DNA sequence) and a genome organization similar to that of a previously reported Escherichia phage (Fig. S1) (26).

Genomic comparisons between MLP2 and Escherichia phages showed a unique DNA region containing a predicted homing endonuclease (HE) that was encoded in MLP2 but absent from those Escherichia phages (Fig. S2). These proteins are site-specific DNA endonucleases that are located in intergenic regions of some phages and represent a significant source of genetic variation between genomes (27). Altogether, our results showed that MLP1, MLP2, and MLP3 had unique genomic regions/ORFs compared to their closest phylogenetic relatives. This suggested that these phages were in fact novel.

### Morphological characterization and host range analysis.

**FIG 2:**
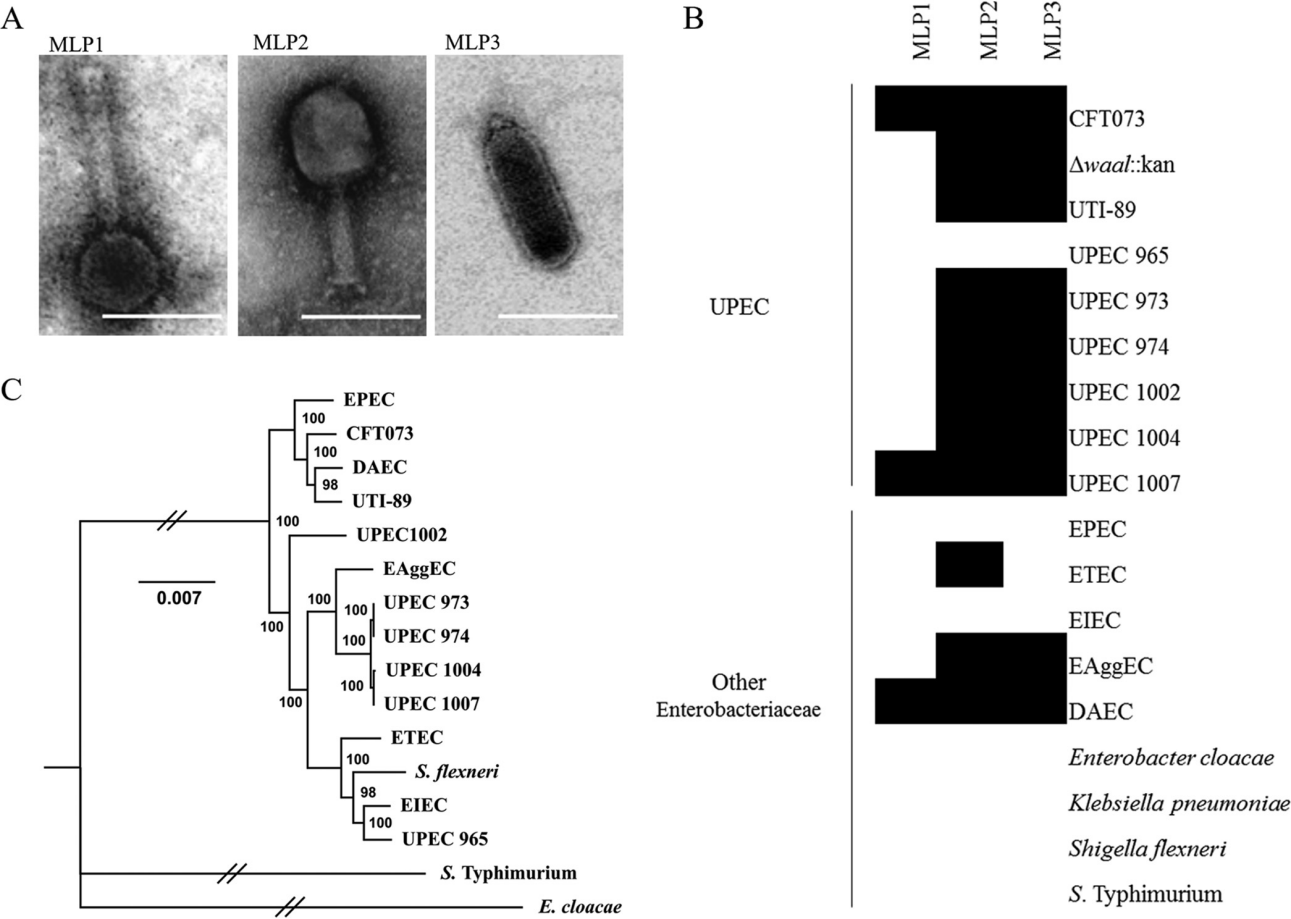
Morphologic and host range analyses of different pathogens. (A) Transmission electron micrographs of MLP phages. The size bars correspond to 100 nm. (B) Host ranges of MLP1, MLP2 and MLP3 among pathogenic E. coli strains were evaluated based on the presence (black) or absence (white) of lysis plaques. (C) Maximum-likelihood whole-genome phylogenetic analysis of all strains used in this study. Bootstrap support is shown for each node.

Transmission electron microscopy (TEM) was used to determine the morphological traits of these phages. Using the ImageJ software (28) for image analysis, we determined that MLP1 had an icosahedral head with an estimated diameter of 66 ± 0.4 nm (mean ± standard deviation) and a tail of 135 ± 0.9 nm in length and 18 ± 0.1 nm in diameter. Similarly, MLP2 had an icosahedral head with an estimated diameter of 63 ± 0.5 nm and a tail of 73 ± 0.7 nm in length and 18 ± 0.7 nm in diameter. MLP3 showed an elongated head of 122 ± 0.5 nm in length and 48 ± 0.4 n in diameter and a tail of 11 ± 0.9 nm in length (Fig. 2A). These results further corroborate our genomic analyses (Fig. 1), since phages corresponding to the *Myoviridae* family display similar morphological traits (29). Likewise, MLP3 exhibited an elongated head and a short tail, which has been defined as a C3 morphology for the *Podoviridae* family (30).

To establish the host range of MLP1, MLP2, and MLP3, we tested them on different laboratory reference strains and clinical isolates of E. coli from patients with diagnosed UTIs. Our results showed that MLP1 had the narrowest host range, killing only CFT073 and the UPEC 1007 clinical isolate. Interestingly, this phage was also able to infect a diffusely adherent E. coli (DAEC) strain that is considered a potential diarrheagenic pathogen (Fig. 2B) (31). In contrast, even though MLP2 and MLP3 were phylogenetically different and belonged to different families (*Myoviridae* and *Podoviridae*, respectively), they exhibited similar host ranges. Both phages were able to infect strain CFT073, strain UTI-89, five UPEC clinical isolates, an enteroaggregative Escherichia coli (EAggEC) strain, and a DAEC strain (Fig. 2B). These results strongly suggested an intestinal origin of MLP2 and MLP3. As stated above, UPEC strains reside in the human intestine. In addition, the host microbiota generates selective pressure, leading to modification of the host range of gut-resident bacteria, which could have led MLP2 and MLP3 to infect E. coli strains colonizing the urinary tract (32). Of note, none of the MLP phages were able to infect other bacterial species, such as Enterobacter, Klebsiella, Shigella, and Salmonella species.

To understand the nature of the clinical strains used in the host range assay and to explain why MLP phages could infect intestinal E. coli strains, we constructed a whole-genome-sequence-based phylogenic tree (Fig. 2C). Our results indicated that DAEC and EAggEC were phylogenetically closer to UPEC than to enterotoxigenic E. coli (ETEC) and enteroinvasive E. coli (EIEC). This might explain the ability of MLP1, MLP2, and MLP3 to infect them (Fig. 2B). Despite the enteropathogenic E. coli (EPEC) strain (Fig. 2C) being phylogenetically closer to the UPEC strains, MLP phages were unable to infect the EPEC strain (Fig. 2B). These findings might be explained by the minimal phylogenetic differences between extraintestinal pathogenic E. coli (ExPEC), InPEC, and commensal E. coli, which mostly rely on the presence of virulence factors encoded in mobile genetic elements (33), by differences in genes encoding phage receptors, and/or by specific mechanisms to avoid phage infection (34, 35).

Based on our genomic analyses, we believe that isolates UPEC 973 and UPEC 974 are most likely the same strain (Fig. 2C). These strains were isolated from two different patients, suggesting that this isolate might be more prevalent in this area. Also, UPEC 965, in spite of being isolated from a UTI patient, was phylogenetically closer to EIEC and Shigella flexneri (Fig. 2C). These results might explain the inability of the MLP phages to infect UPEC 965 (Fig. 2B). To further characterize the UPEC clinical isolates used in this study, we used ResFinder to determine the presence of antibiotic resistance genes (36). We observed that most of the UPEC isolates contained antibiotic resistance for at least 2 antibiotics due to either the presence of antibiotic resistance genes or point mutations (Table S2). These results not only supported the complex scenario of antibiotic resistance but also highlighted the ability of these novel phages to effectively kill the current MDR strains associated with UTIs.

### EOP and killing dynamics on E. coli strains.

**FIG 3:**
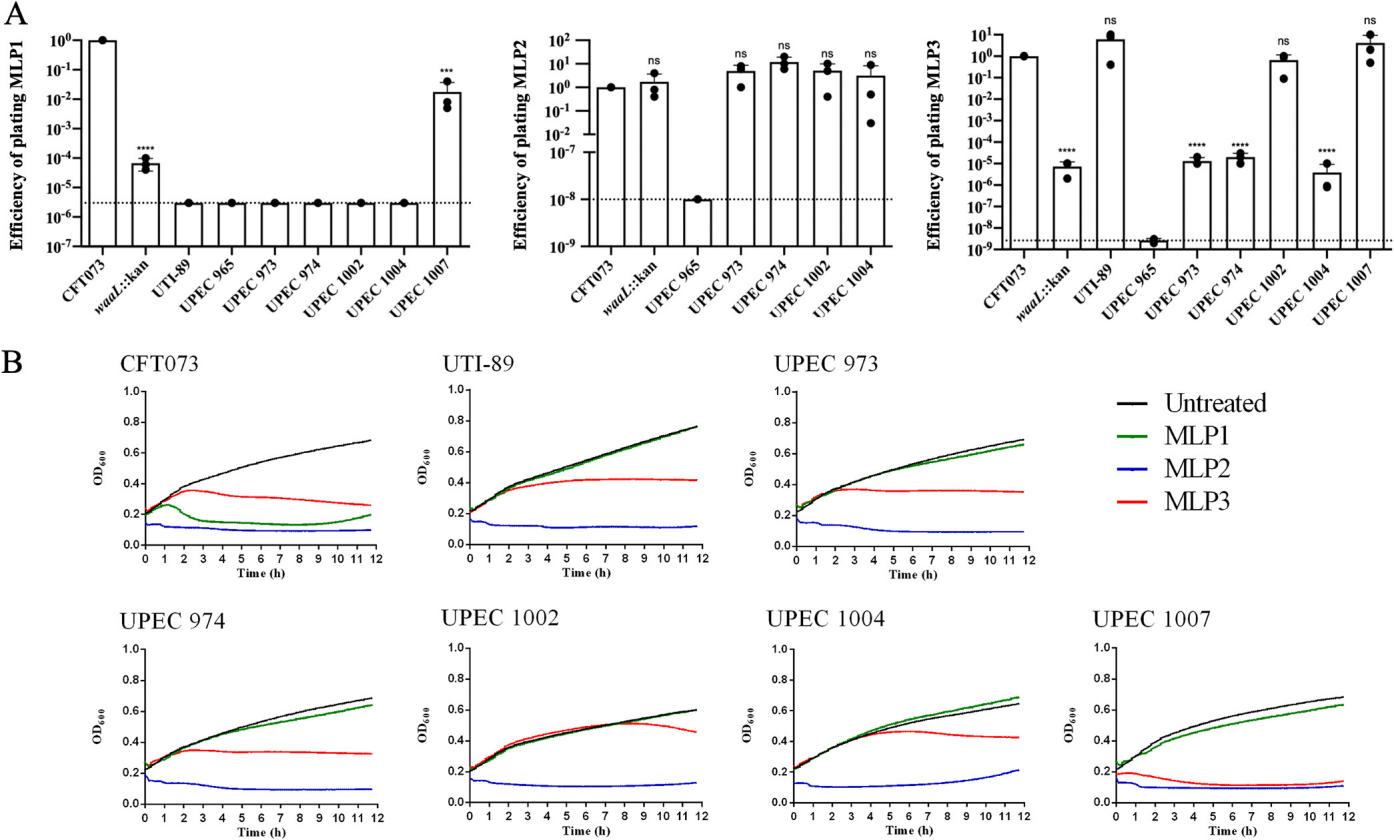
Efficiency of plating (EOP) and killing dynamics of UPEC strains exposed to MLP phages. (A) EOP was determined in UPEC cultures challenged with MLP phages (MOI of >1). EOP was calculated as the ratio between the titer of each phage on a test strain and the titer on CFT073 wild type (*n* = 3). Dotted lines represent the limits of detection of the assay. Statistical significance was determined using the two-tailed Student’s *t* test: *, *P* < 0.05; **, *P* < 0.01; ***, *P* < 0.001; ****, *P* < 0.0001; ns, not significant. (B) Growth measurements for bacterial cultures treated with MLP1, MLP2, MLP3, and the untreated control. Curves represent the mean values from 4 independent experiments.

To determine the efficiency of these phages in killing the different strains tested for their host range, we determined the efficiency of plating (EOP) on pathogenic E. coli strains. Our results showed that MLP1, as seen in the host range assay, was only able to infect the UPEC 1007 clinical isolate with ∼100-fold lower efficiency than it showed for infection of CFT073 (Fig. 3A). Moreover, confirming this result, MLP1 plaques were significantly smaller after infection of UPEC 1007 than after infection of CFT073 (Fig. S3). In addition, an ∼4-log decrease in EOP was observed for MLP1 against the CFT073 Δ*waaL* strain, confirming the data from the host range assay suggesting that the O antigen is a receptor for MLP1 (Fig. 3A). The *waaL* gene encodes an O-antigen ligase (37). Mutants with an inactivated *waaL* gene cannot complete the lipopolysaccharide (LPS) biosynthesis pathway, and thus, they lack the surface-exposed portion of O-antigen molecules.

LPS is an abundant, immunogenic macromolecule anchored in the outer membrane of Gram-negative bacteria. This molecule consists of three main structural domains: lipid A, embedded in the outer membrane, the core oligosaccharide (inner and outer), and the O antigen, which is the distal portion exposed on the bacterial surface. Lipid A and the inner core are highly conserved among bacteria, whereas the O antigen is the most variable region of the molecule even among serotypes from the same bacterial species. It has been previously reported that the O antigen is the receptor that some phages utilize for recognition on the cell surface prior to infection (38). MLP1 infection of the CFT073 Δ*waaL* strain produced only a couple of detectable viral particles (Fig. S3). However, the absence of *waaL* did not completely abolish MLP1 replication.

Contrary to the results for MLP1, MLP2 showed similar efficiencies of plating between CFT073, CFT073 Δ*waaL*, and the UPEC clinical isolates 973, 974, 1002, and 1004 (Fig. 3A). These findings were confirmed by MLP2-dependent efficient killing, as observed by lack of growth of these strains in the presence of MLP2 (Fig. 3B). Interestingly, small turbid plaques were observed for MLP2 when infecting all the tested strains. Since the predicted lifestyle of MLP2 is to carry out lytic cycles (Fig. 1A), the turbid plaques observed might have been a consequence of an impaired/impeded ability to generate plaques mediated by the composition of the medium, as has been demonstrated for other phages (39), or by bacterial glycosylation of LPS, which has been proposed as a mechanism that may impact cell sensitivity to phages (40). In the case of UTI-89 and UPEC 1007, we were not able to determine isolated PFU after MLP2 infection, which might be attributed to an LB agar-dependent interference in the ability of MLP2 to generate plaques, as was mentioned above. However, efficient MLP2-mediated killing of UTI-89 and UPEC 1007 was observed by growth curves (Fig. 3B) and by its ability to impact bacterial survival (Fig. S4).

The host range profile of MLP3 seemed to be similar to that of MLP2 (Fig. 2B). However, the EOP of MLP3 varied among UPEC strains. MLP3 killed UTI-89, UPEC 1002, and UPEC 1007 efficiently compared to its killing of the CFT073 control. A 5-log reduction in EOP was observed for CFT073 Δ*waaL* and UPEC strains 973, 974, and 1004 (Fig. 3A). This also suggested, as seen for MLP1, that the O antigen was involved in the recognition by MLP3. Additionally, it indicated that these clinical isolates might have lacked the receptor for MLP3 but carried a coreceptor that allowed infection with much lower efficiency.

Growth curves for these UPEC strains in the presence of MLP3 showed that UTI-89 was able to grow for 2 h before its growth was arrested (Fig. 3B). Conversely, MLP3 generated an early arrest of growth in UPEC 1007 (Fig. 3B), even when it displayed an EOP similar to its EOP on UTI-89 (Fig. 3A). Altogether, these results showed that MLP1, MLP2, and MLP3 displayed different killing dynamics depending the UPEC host they infected.

**FIG 4:**
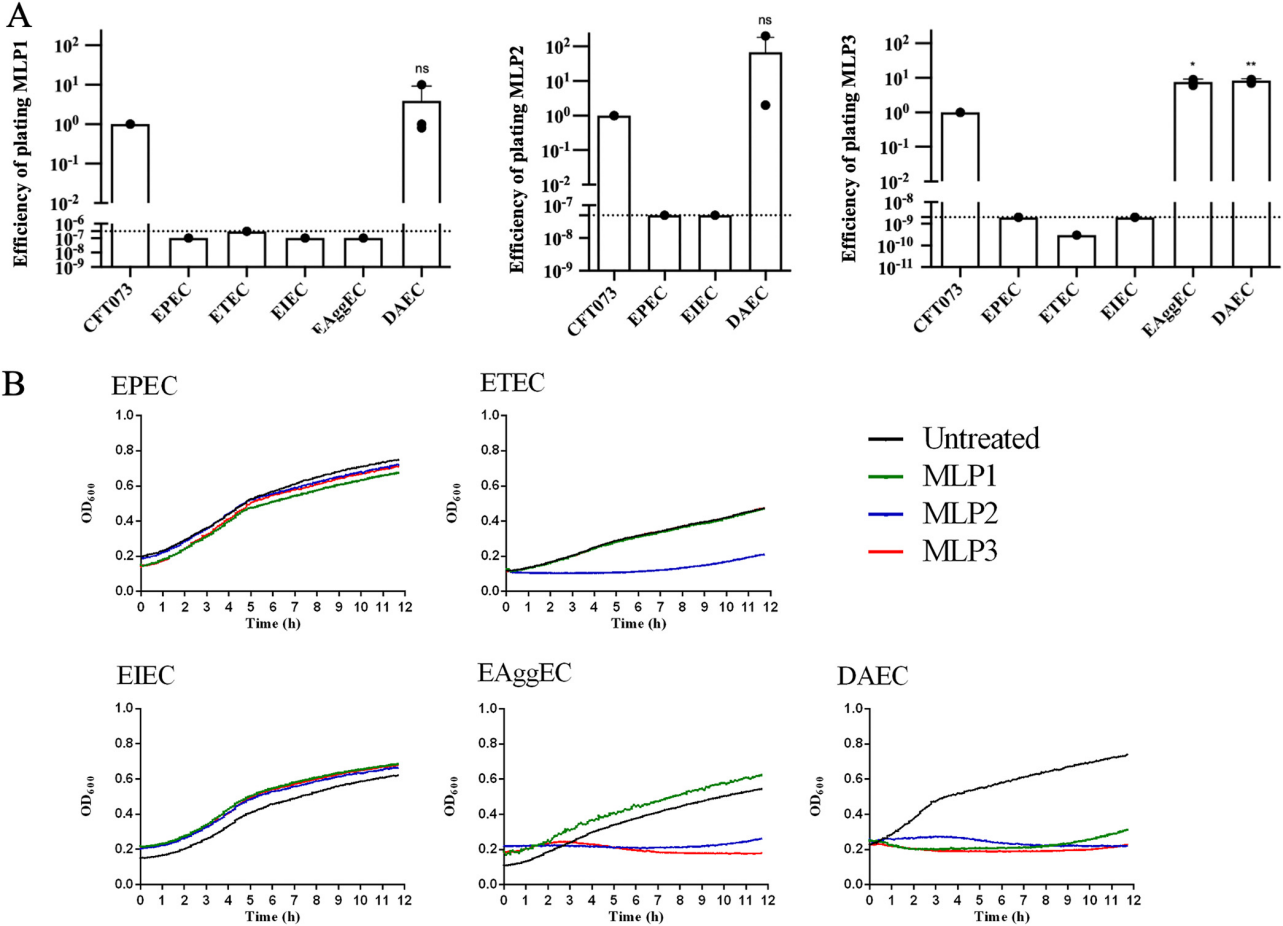
Efficiency of plating (EOP) and killing dynamics of InPEC strains exposed to phages. (A) EOP was determined in cultures exposed to MLP phages (MOI of >1) (*n* = 3). Dotted lines represent the limits of detection of the assay. Statistical significance was determined using the two-tailed Student’s *t* test: *, *P* < 0.05; **, *P* < 0.01; ***, *P* < 0.001; ****, *P* < 0.0001; ns, not significant. (B) Growth measurements in the presence of MLP1, MLP2, MLP3, and the untreated control. Curves represent the mean values from 4 independent experiments.

Analysis of EOP on InPEC strains showed that all phages killed diffusely adherent E. coli (DAEC) efficiently (Fig. 4A), sometimes to an even greater extent than CFT073, whereas only MLP2 and MLP3 could kill EAggEC, as evidenced by their impacts on its growth ability (Fig. 4B). It is important to note that both DAEC and EAggEC have been shown to cause UTIs (41, 42), where they could be exposed to the MLP phages.

We were not able to obtain isolated PFU in EOP assays for MLP2 on ETEC and EAggEC (Fig. S3). However, growth curves (Fig. 4B) and bacterial survival assays (Fig. S4) in the presence of this phage showed that MLP2 was in fact able to kill these strains.

### Phage receptor identification.

Based on EOP and host range assays, we identified the O antigen as the main receptor needed by MLP1 and MLP3 for efficient infection. However, phage replication was not completely impaired on the CFT073 Δ*waaL* mutant (Fig. 3A). To evaluate whether the O antigen was indeed the receptor for these phages and to identify possible coreceptors for MLP1 and MLP3 and the receptor for MLP2, we exposed CFT073 bacterial cultures to each of the MLP phages. After overnight incubation, resistant mutants were isolated and their genomes were subjected to whole-genome sequencing (WGS) for variant detection analyses. After infection, turbid plaques were generated by MLP2 and MLP3 in these mutants, suggesting that the LPS molecule might act as a coreceptor (43). Similar EOP values were observed for MLP1 and MLP3, indicating that O antigen was critical for recognition of MLP1 and acted as its main receptor (Fig. 3A).

**FIG 5:**
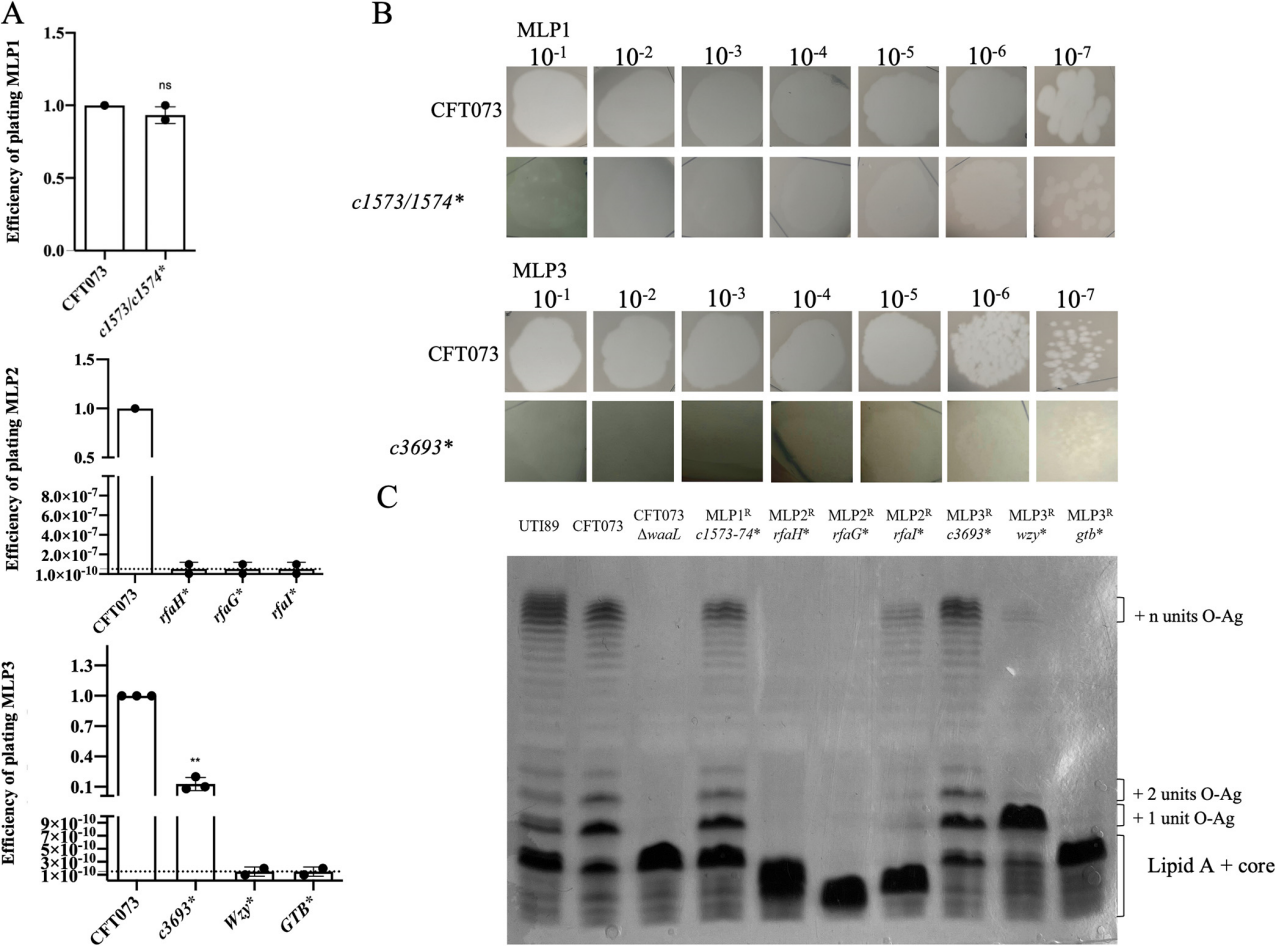
Characterization of phage-resistant E. coli mutants. The efficiencies of plating (EOPs) of MLP phages (A), morphology of plaques (B), and LPS profiles of variants (C) were determined on isolated resistant mutants. O-Ag, O antigen.

By WGS and variant analysis of five different MLP1-resistant clones, we identified two resistant colonies carrying mutations in ORFs *c1573* and *c1574* (c1573/c1574*), encoding lambda head decoration protein D, which is a predicted capsid shell-stabilizing protein (Table 1). Interestingly, MLP1-mediated killing on the c1573/c1574* mutant showed no statistical difference from the MLP1-mediated killing on the wild-type strain (Fig. 5A). However, infection of the c1573/c1574* strain resulted in turbid plaques (Fig. 5B). Also, the c1573/c1574* mutant showed an LPS profile similar to that of the CFT073 wild type (Fig. 5C), suggesting there were no additional mutations in the O antigen that could lead to MLP1 resistance.

**TABLE 1:** Identification of phage receptors by whole-genome sequencing analysis of phage-resistant E. coli mutants

Phage to which isolate is resistant	Reference position	Reference	Allele	Overlapping annotations	Amino acid change
MLP1	1430717	CG	GC	*c1574*, bacteriophage lambda head decoration protein D	c1573:p.Val109Leu; c1574:p.His9_Gly10delinsGlnArg
	1430717	CG	GC	*c1574*, bacteriophage lambda head decoration protein D	c1573:p.Val109Leu; c1574:p.His9\?\Gly10delinsGlnArg
MLP2	4237415	GC		*rfaG*, glycosyltransferase	c4455:p.Ala219fs
	4235455	A	C	*rfaI*, lipopolysaccharide 1,3-galactosyltransferase	c4453:p.Leu229*
	4550384	G	T	*rfaH*, transcriptional activator RfaH	c4789:p.Ser97*
	4550619	C	A	*rfaH*, transcriptional activator RfaH	c4789:p.Glu19*
MLP3	2397883		T	*c2564*, O-antigen polysaccharide polymerase Wzy	c2564:p.Asn404fs
	2393040	C		*c2559*, glycosyltransferase_GTB_type	c2559:p.Val178fs
	3521953		T	*c3693*, XcbB/CpsF family capsular polysaccharide biosynthesis protein	c3693:p.Phe95fs
	3521953	T		*c3693* XcbB/CpsF family capsular polysaccharide biosynthesis protein	c3693:p.Lys94fs

Altogether, these data suggested that lambda head decoration protein D might compete with viral proteins during the assembly of the capsid. Structural differences from the native proteins of MLP2 might generate viral particles with fitness defects that might explain the generation of turbid plaques.

Analysis of 5 independent MLP2-resistant clones showed mutations in four isolates that were traced to different regions of the LPS molecule. These mapped to the *rfaG* (*waaG*) and *rfaI* (*waaO*) genes, encoding proteins involved in the LPS outer core biosynthesis pathway (Fig. S5) (44). Also, two different variants were identified in the *rfaH* (also named *hlyT* and *sfrB*) gene, encoding a transcriptional regulator that participates in the assembly of the LPS outer core (Table 1) (45). No plaques were observed in cultures of any of these resistant mutants after exposure to MLP2 (Fig. 5A), indicating that indeed the MLP2 receptor was the LPS outer core. In agreement with the observed MLP2-resistant phenotype and our genomic-variant analyses, the LPS profiles for the rfaH* and rfaG* variants displayed the profile of a deep rough mutant with an incomplete core molecule. This was observed by faster migration of the LPS core than for the CFT073 wild type. Conversely, the LPS profile of the rfaI* variant also showed faster migration of the LPS core. However, some O-antigen polymerization was seen (Fig. 5C). These results might be explained by a modification in the activity of RfaI that did not inactivate the enzyme, leading to changes in the O-antigen structure (Fig. S5).

Regarding MLP3-resistant mutants, variants in the *c2564*, *c2559*, and *c3693* genes were identified (Table 1). These genes encode the O-antigen polysaccharide polymerase Wzy, the glycosyltransferase (GT) GTB, and the XcbB/CpsF family capsular polysaccharide biosynthesis protein, respectively. Both GTB and Wzy belong to the LPS Wzx/Wzy-dependent assembly pathway, the main O-antigen biosynthetic pathway (46, 47). Specifically, GT completes the carbohydrate structure, adding repeat sugar units to undecaprenyl diphosphate (Und-PP), which is a molecule that allows the assembly of O antigen at the cytoplasmic face of the inner membrane. Then, O-antigen repeat units are flipped at the periplasm by the Wzx protein and assembled by the Wzy polymerase (Fig. S5) (46, 47).

XcbB/CpsF belongs to a capsule gene cluster that encodes enzymes involved in the synthesis of a capsular polysaccharide (48, 49). EOP assays of MLP3 on the resistant strains showed that MLP3 was not able to infect the wzy* and gtb* variants. Accordingly, the LPS profiles of the wzy* and gtb* variants were indicative of a structure of lipid A-core moiety with one and no O-antigen sugars, respectively (Fig. 5C). On the other hand, MLP3 was able to infect the c3693* variant with an ∼90% lower efficiency than for CFT073 (Fig. 5A), also generating a smaller and turbid plaque (Fig. 5B). In addition, these results further confirm our previous findings and indicate that the receptor of MLP3 was the polymerized O antigen and the capsular exopolysaccharide was used by the phage as a coreceptor. A global model for the interactions of phages with their receptors is summarized in Fig. S5.

## DISCUSSION

The use of bacteriophages as an alternative approach for the treatment of bacterial infections was proposed more than 100 years ago. However, with the discovery of antibiotics, phage therapy was neglected and not further explored except for some countries in Eastern Europe (50). The antibiotic resistance crisis demands the generation of novel and/or complimentary antibacterial strategies. In this context, the use of specific and personalized phage cocktails has proven to be game-changing in the war against MDR bacteria (51). For this, constant isolation of novel phages able to kill the actual antibiotic-resistant pathogens and a comprehensive understanding of the biology of these phages are essential to develop novel antibacterial strategies that might be used to treat human pathogens.

In this work, we isolated 3 novel phages, MLP1, MLP2, and MLP3, using UPEC CFT073 as a bacterial host. This strain was isolated from a patient with acute pyelonephritis 25 years ago (52). Our genomic analyses show that MLP2 has a higher number of encoded tRNAs than MLP1 and MLP3 (Fig. 1A) and that a unique sequence of MLP2 encodes a predicted homing endonuclease that is absent from the 5 most-closely related phage genomes (Fig. S2). With these findings, we can hypothesize that MLP2 may have coacquired HE and viral tRNAs, as has been suggested previously (53). This phage may have been exposed to selective pressures generating an impact on its fitness, which might explain its broader host range as evidenced by its ability to infect intestinal pathogenic E. coli. This hypothesis is supported in particular by our findings showing that ETEC strain 10407 is infected exclusively by MLP2 (Fig. 2B).

MLP1 belongs to the *Chaseviridae* family as evidenced by phylogenetic analysis. However, compared to MLP2 and MLP3, it exhibits a more restricted pattern of host recognition (Fig. 1C and 2B, respectively). This difference in the host range seems to be related to the recognition of different regions of the LPS molecule, as evidenced by (i) the ability to infect a Δ*waaL* strain (Fig. 2B) and (ii) variant analysis of phage-resistant mutants showing the role of different steps of the LPS biosynthesis process for an efficient infection for all MLP phages (Table 1). These results suggest that MLP1 might recognize a specific sugar or a stoichiometric structure in the O-antigen repeat that is not common among UPEC strains and is also present in some InPECs with potential to cause UTI infections (DAEC).

**FIG 6:**
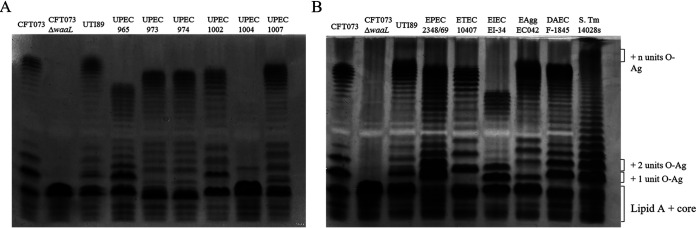
LPS profiles of pathogens used in this study. LPS profiles of UPEC (A) and InPEC strains (B) were analyzed by Tricine-SDS-PAGE. O-Ag, O antigen.

MLP3 belongs to the *Podoviridae* family, and its host range is highly similar to that of the MLP2 phage (Fig. 2B). This is particularly interesting considering that MLP2 recognizes the conserved LPS outer core and MLP3 interacts with the highly variable O antigen as its main receptor. Despite being able to infect mostly the same pathogenic E. coli strains, the efficiencies in the infection cycles differed considerable between MLP2 and MLP3, and this could only be evidenced by EOP assays. MLP2, which recognizes a structure that is more conserved among strains of the same species, also showed the same level of killing for all the UPEC strains tested (Fig. 3). In agreement with this line of thought, MLP3, which recognizes a highly variable structure in the LPS molecule, showed high variability in its killing profiles for all the UPEC strains that were tested (Fig. 3A). A deeper analysis of LPS profiles (Fig. 6A and B) and gene clusters for LPS and capsule biosynthesis (Fig. S6 and S7, respectively) allowed us a more comprehensive understanding of the abilities of MLP phages to infect pathogenic E. coli. These strains exhibit different LPS profiles and genomic organization of their O-antigen clusters (Fig. S6A), which is consistent with our findings showing similar efficiencies of plating in these pathogens (Fig. 4A).

A very interesting result is that the three MLP phages were able to infect DAEC to the same or an even greater extent than they could infect CFT073. This is intriguing for MLP1, since it showed a very restrictive host range for successful infection. This suggests that DAEC and CFT073 share similar features in their LPS core (recognized by MLP2) and in the O antigen, which is the structure that can be recognized by MLP1 and MLP3. A comparison of the O antigen between CFT073 and DAEC shows high variability (Fig. S6A). In contrast, a comparison of both the O antigen and the LPS core clusters shows that the genomic organization of the LPS core (recognized by MLP2) and the colanic acid capsule polysaccharide (recognized by MLP3) is highly conserved between these two strains (Fig. S6B and S7A). However, some conserved genes are shared between CFT073 and DAEC. The gene products from these genes could be crucial for the biosynthesis of the common structure that is recognized by the MLP1 and MLP3 phages.

The variation between bacterial O antigens among bacterial serotypes and species is the result of multiple selective pressures, such as bacteriophage predation and the action of the immune system. This variability confers an adaptive advantage to bacteria in a given specific niche; e.g., loss of LPS is associated with a decreased virulence (54, 55). Despite the diversity of O antigen and core structures of bacterial LPS, bacteriophages can evolve to infect different hosts but remain specific for the same bacterial species (56). In the case of the MLP phages, they were able to infect pathogenic E. coli strains that shared similar structures in their LPS. However, none of the MLP phages could infect other bacterial species, such as Enterobacter, Salmonella, Klebsiella, and Shigella. In addition, phage-resistant mutants may become avirulent and/or trigger the immune response (57), which advances the notion of phage therapy or the use of phages as complementary therapeutic agents along with current antimicrobials.

None of the MLP phages were able to infect the UPEC 965 clinical isolate. When comparing the O-antigen clusters from all the UPEC strains tested (Fig. S6A), this strain shows a highly distinctive gene content and organization. One of the genes that is only present in UPEC 965 is *gla*, which encodes a UDP-galacturonatenase (Fig. S6A) (54). This suggests that the UPEC 965 isolate might carry a modification exposing a UDP-GalA sugar to the surface that cannot be recognized by the MLP phages. The unique arrangement of the O-antigen cluster in UPEC 965 agrees with the LPS profile, where this strain differs from all the other UPEC strains tested (Fig. 6). Of note, the MLP2 receptor is the LPS core and not the O antigen, but this phage was also unable to infect UPEC 965. This could be due to an interruption in the *waaI* gene that was only present in UPEC 965 among all the UPEC strains from this study (Fig. S6B).

UPEC 1004, which is susceptible to MLP2 and MLP3, also showed a strikingly different LPS profile than the rest of the UPEC clinical isolates from this study (Fig. 6). Moreover, this strain seems to expose just one unit of O antigen. In fact, the lack of O-antigen repeats exposed to the surface (Fig. 6A) seems to be a critical factor for MLP3 infection, since the EOP when infecting UPEC 1004 was 5 log lower than the EOPs for CFT073, UTI-89, UPEC 1002, and UPEC 1007 (Fig. 3). However, these strains still carry the *xcbB* gene (Fig. S6B), which by acting as coreceptor for MLP3, might explain the only partial reduction in the ability to generate plaques (Fig. 3A). Similarly, UPEC 973 and 974 were also infected with lower efficiency by MLP3. However, both strains carry the *wbuC* gene, which is absent from every other UPEC isolate and has been proposed to be a gene remnant (58). This might affect the LPS biosynthesis pathway or generate a lower accessibility of its receptor to MLP3.

When comparing LPS cluster gene contents and arrangements for UPEC strains (Fig. S6), UPEC 1007 is highly similar to UPEC 1004. Interestingly, UPEC 1007 is one of the few isolates that can be infected by MPL1. The only difference between the UPEC 1004 and 1007 O antigens is the interruption of the *manC* gene by the insertion of the *rfbM* gene. This observation strongly suggests that the *manC* product (GDP-Man) (54) could be the sugar recognized by MLP1. On the other hand, EggAgEC displays the same genomic arrangement for the O antigen as 1007, but it is not susceptible to MLP1. This could be due not to the recognition of the O1 sugar exposed to the surface but to single nucleotide variations in *c1573/1574* that were shown to have a role in the MLP1 infective cycle (Table 1 and Fig. 5).

Finally, although EPEC is phylogenetically closer to UPEC strains CFT073 and UTI-89 (Fig. 2C), none of the phages were able to infect this strain (Fig. 2B). Most likely, this is a consequence of the vast differences in its LPS profile (Fig. 6B and Fig. S6). In the particular case of MLP3, since EPEC does not have a capsule, the absence of the coreceptor may also impede phage infection.

Altogether, our data point to a constant arms race between phages and bacteria. In this context, the MLP phages have been shown to be able to infect both the CFT073 and UTI-89 strains that were isolated 20 years ago from a patient with UTI (59) and current MDR UPEC isolates with similar efficiencies. These results suggest that MLP1, MLP2, and MLP3 are coevolving with bacteria to keep their infectivity. In addition, since DAEC and EAggEC are able to generate UTIs (41, 42), we hypothesize that the selective pressures generated by phages might allow E. coli to evolve, leading it to change its niche in order to avoid phage predation. This could be the case for these InPEC strains, which can infect the urinary tract as well. Concomitantly, *in vitro* phage predation dynamics may be used as a readout to track these evolutionary traits of phages and bacteria from the past and the present. These studies are currently being conducted in our laboratory.

The novel MLP1, MLP2, and MLP3 phages isolated here are able to infect MDR InPEC strains by recognizing different regions of LPS. The LPS molecule is a known virulence factor in UPEC (60), and it has been demonstrated that LPS mutants of UPEC strains have an impaired ability to generate bacterial reservoirs in bladders and an increased humoral response in animal models (57). Altogether, these data render feasible the use of novel bacteriophages MLP1, MLP2, and MLP3 as potential therapeutic alternatives.

## MATERIALS AND METHODS

### Bacterial strains and growth conditions.

Cultures of CFT073, CFT073 Δ*waaL*, UTI-89, MDR clinical isolates UPEC 973, 974, 1002, 1004, and 1007, EPEC 2348/69, ETEC H10407, EIEC EI-34, EAggEC 042, DAEC F-1845, Enterobacter cloacae ATCC 23355, Shigella flexneri 2457T, and Salmonella Typhimurium 14028s (Table S1) were routinely grown in Luria-Bertani (LB) broth at 37°C with vigorous shaking.

Clinical isolates of UPEC were isolated from patients with UTIs in the city of Valdivia, Chile. Informed consent was obtained from patients allowing the use of samples for this study. Intestinal pathogenic E. coli strains were kindly provided by Roberto Vidal (Facultad de Medicina, Universidad de Chile).

### Phage isolation.

Water samples were collected in 2019 from the Valdivia River in the city of Valdivia, Chile. Samples were centrifuged to remove large particles and filtered using 0.22-μm-pore-size filters. To enrich phages in the sample, an overnight (ON) culture of UPEC CFT073 was diluted in 50 mL of LB (1:250 dilution) and incubated for 1 h at 37°C with shaking. Then, a 1:1 mixture of culture and filtered sample was prepared and incubated ON at room temperature (RT). The next day, cultures were centrifuged, filter sterilized, and serially diluted, and 10-μl aliquots were spotted on top LB-agarose overlays (0.3% [wt/vol] agarose in LB broth) containing 200 μl of an ON culture of CFT073. After ON incubation at RT, isolated plaques displaying different morphologies were isolated and serially purified on top LB-agarose overlays as mentioned above.

### Titer enrichment of isolated bacteriophages.

High-titer stocks of phages were prepared as described previously (17). Briefly, 4 mL of top agar was mixed with 50 μl of the primary phage stock, 200 μl of an ON culture of CFT073, and laid over LB-agar plates. After ON incubation at RT, the top agar was harvested, suspended in 3 mL of phage buffer (100 mM NaCl, 10 mM Tris-HCl [pH 8.0], 10 mM MgSO_4_), and vortexed vigorously for 2 min. The lysates were centrifuged for 10 min at 4,750 rpm, and the supernatants were filter sterilized and stored at 4°C. Phage titers were determined by PFU counting.

### Genome sequencing and assembly.

Phage genomic DNA was purified using the phage DNA isolation kit (Norgen Biotek) following the recommendations of the manufacturer. Molecular libraries for deep sequencing were prepared using the Nextera XT DNA library preparation kit (Illumina). Samples were pooled and purified using QIAquick PCR DNA cleanup (Qiagen). A single-end 100-bp high-output sequencing run was conducted using the Illumina HiSeq 2500 at the Tufts University Core Facility (TUCF Genomics).

High-quality draft genomes were assembled *de novo* using the CLC Genomics Workbench 8.1 software (Qiagen). Assembly was filtered to contain contigs of ≥1,000 bp. For detection of antibiotic resistance genes, raw Illumina reads were submitted to Resfinder 4.0 at the Center for Genomic Epidemiology (24, 36, 61, 62). Phage genomes were assembled into a single contig and then annotated using RAST (63–65).

### Phage genome annotation.

Genomes were initially predicted and annotated using the RAST server, as mentioned. Then, to improve the annotation, protein-coding sequences were translated, analyzed using the BLASTp tool (24), and compared against the viral subset of the RefSeq database release 205 (66), the Swiss-Prot database (67), the Virulence Factor Database (VFDB) (68), and the Prokaryotic Virus Orthologous Groups (pVOGs) database (69). Hits with an e value smaller than 10^−7^ were selected for further analysis. Also, a protein motif search was conducted using the hmmscan tool embedded in HMMER version 3.1 (70) and compared with the Pfam (71) and TIGRfam (72) databases. Putative structural proteins, Rho-independent transcription terminators, tRNAs, and promoters were predicted using PhaNNs (73), ARNold (74), the tRNAscan-SE tool (75) and the PhagePromoter tool (76), respectively.

### Phylogenetic analyses.

Taxonomic classification of phages was assessed by a proteome-based tree using the VipTree platform (21). Amino acid sequences of terminase large subunit (TerL) proteins from related phages were aligned with the Clustal Omega tool (77) and manually trimmed.

Alignments were used to construct maximum-likelihood phylogenetic trees using PhyML version 3 (78) using 1,000 bootstrap replications. ProtTest version 3.42 software was used to choose the evolutionary model for each reconstruction as described previously (79).

Phylogenomic analyses of E. coli strains and isolates were done using the REALPHY software, using the CFT073 and UTI-89 strains as references. The trees were inferred via PhyML, with 1,000 bootstrap replications (80).

### Electron microscopy.

Phage lysates (50 μl) were added onto a Formvar/carbon-supported copper grids and stained with 2% aqueous uranyl acetate for 30 s. Transmission electron micrographs were obtained using a Libra 120 plus transmission electron microscope (Zeiss) at 80 kV at the Austral University (UACH) core microscopy facility.

### Host range determination.

The ability to infect different enteric bacteria was determined by plaque assays (Fig. 3). Briefly, strains were cultured to an optical density at 600 nm (OD_600_) of ∼0.1 and aliquots were mixed with 4 mL of top agarose and laid over LB agar plates. Ten microliters of a high-titer phage stock was deposited onto the top agarose overlays. Plates were incubated ON at RT and examined for the ability to generate lysis plaques.

### EOP.

The efficiency with which the phages could infect E. coli strains was assayed by spot titer assays. Briefly, strains were grown ON, and aliquots were mixed with 4 mL of top agarose and laid over LB agar plates (typically, 4 × 10^8^ cells were added). Ten-microliter amounts of serial 10-fold dilutions of phages were spotted onto the top agarose overlays. The plates were incubated ON at RT, and the titer were determined by the generation of lysis plaques. The efficiency of plating (EOP) was calculated by dividing the titer of each phage on the test strain by the titer on its isolation strain (CFT073).

### Growth curves.

Monitoring of growth curves was performed using a Tecan M nano+ plate reader. Bacterial cultures were grown to an OD_600_ of ∼0.1 and infected with phages using a multiplicity of infection (MOI) of >1 (MOI values ranged between 1 and 5). Plates were incubated for 12 h statically at 25°C (the optimal temperature for infection of each phage) during the experiment.

### Identification of phage receptors.

A primary phage stock was mixed with an ON culture of CFT073 culture in overlays of top agarose and laid over LB agar plates. After ON incubation at RT, 5 phage-resistant colonies were picked and streaked on LB agar plates. Single colonies were used to inoculate fresh LB, and after ON growth, liquid cultures from resistant colonies were used for genomic DNA purification using the DNeasy blood and tissue kit (Qiagen) following the instructions of the manufacturer. Sequencing and assembly were performed as described above. Variant analysis was conducted using the CLC Genomics Workbench 8.1 software (Qiagen).

### LPS analysis.

Samples were prepared as previously described (81). Briefly, 1.5-mL amounts of ON bacterial cultures were centrifuged. Then, bacterial pellets were suspended in 100 μL of lysis buffer containing 200 μg/mL proteinase K. LPS was separated using a Tricine-SDS buffer system (Tricine-SDS-PAGE) in 12% (wt/vol) acrylamide gels (82).

### Data availability.

Illumina sequence data for the MLP phages and pathogens used in this study were deposited in the NCBI Sequence Read Archive (SRA) under BioProject accession number PRJNA761370.
