# Supplementary material for: Isolation and Characterization of Novel Lytic Phages Infecting Multidrug-Resistant Escherichia coli
Source: Microbiol Spectr. 2022 Feb 16;10(1):e01678-21. doi: 10.1128/spectrum.01678-21 (PMC8849078; doi:10.1128/spectrum.01678-21)
Supplement: SUPPLEMENTAL FILE 1 — Supplemental material. Download SPECTRUM01678-21_Supp_1_seq1.pdf, PDF file, 6.4 MB [file spectrum01678-21_supp_1_seq1.pdf]

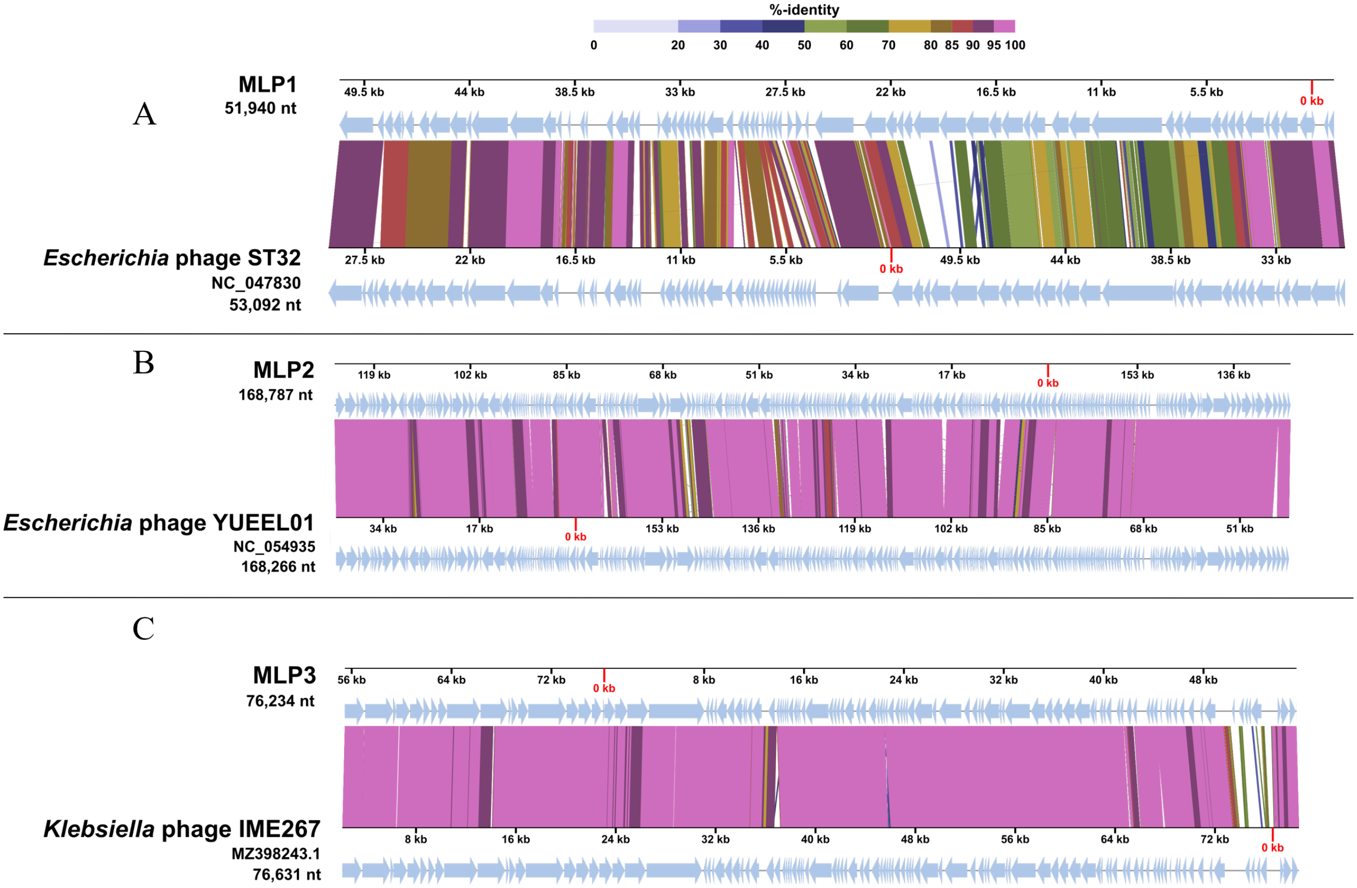

Figure S1. Comparative analyses of MLP phage genomes. Genomes of phages MLP1 (A), MLP2 (B) and MLP3 (C) were aligned and compared with their closest relative found in the nucleotide non-redundant NCBI database.

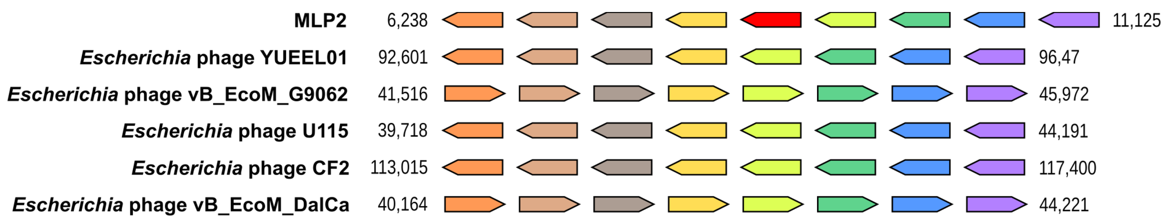

Figure S2. Comparison of the unique region of MLP2 with its most similar phages. The genomic region of MLP2 containing an ORF encoding for a homing endonuclease (red arrow) was aligned with the 5 most similar phages at level of identity. Numbers represent the position in the phage genome.

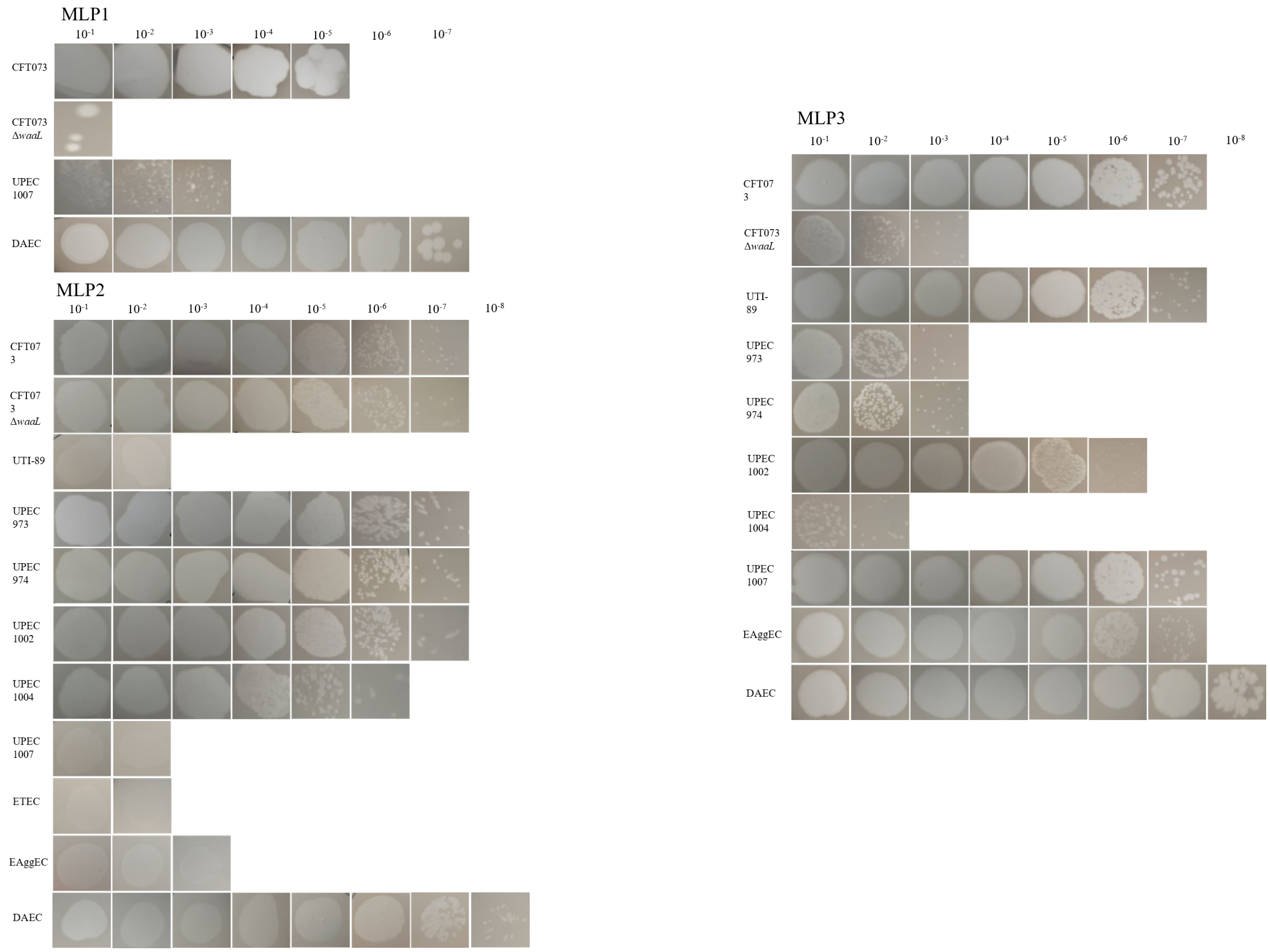

Figure S3. Efficiency of platting (EOP) of phages on *E. coli* strains. Morphology of plaques generated by MLP1, MLP2 and MLP3 are shown.

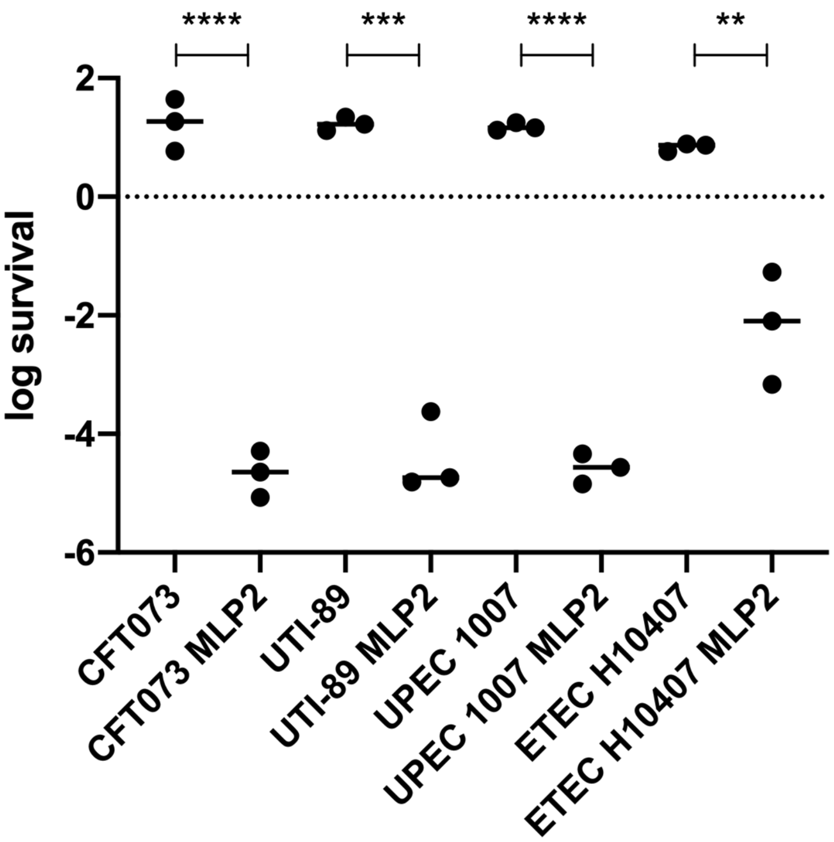

Figure S4. Impact of MLP2 on bacterial survival of pathogenic *E. coli.* The ability of MLP2 to infect and kill pathogenic *E. coli* was addressed by CFU counting (n=3). Statistical significance was determined using a two-tailed Student *t* test (*, *P* 0.05; **, *P* 0.01; ***, *P* 0.001; ****, *P* 0.0001).

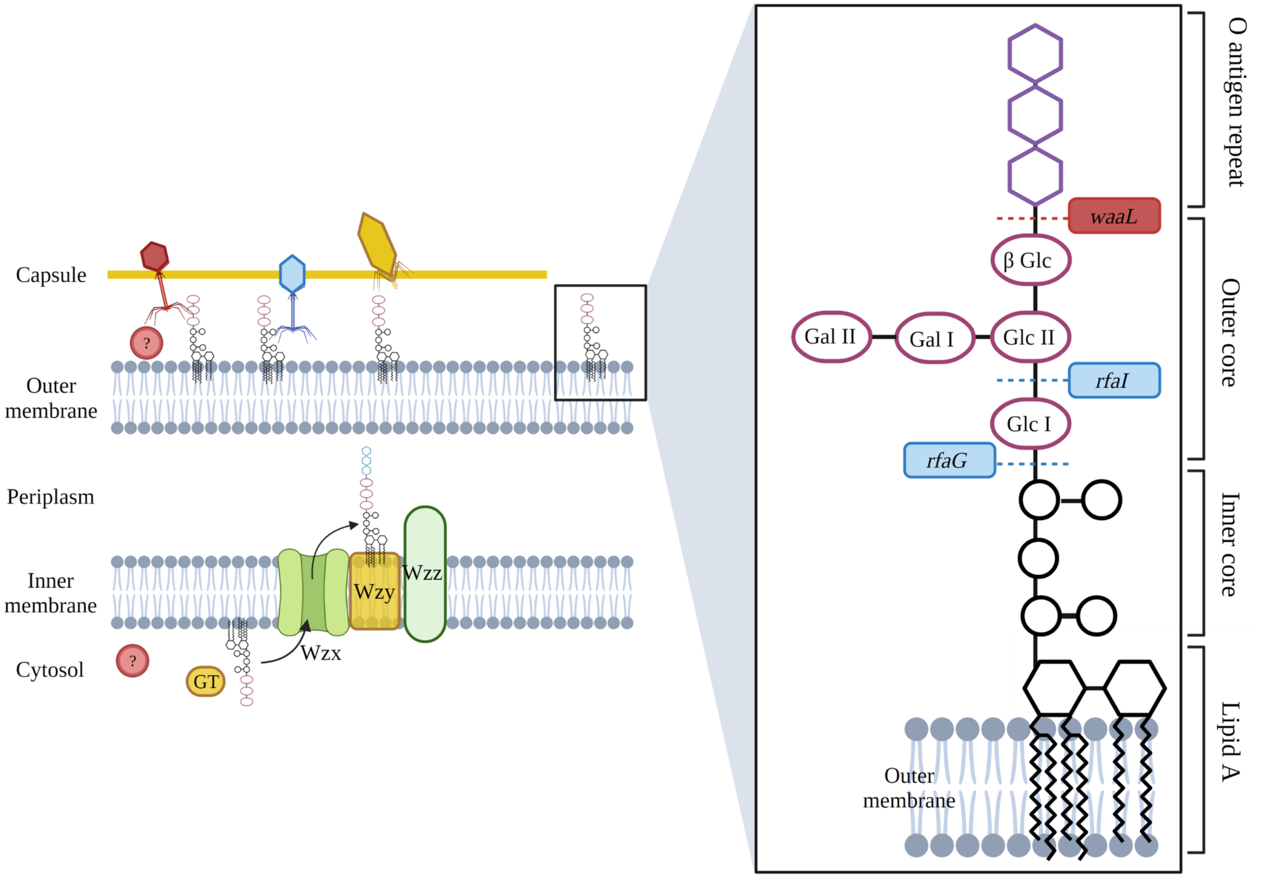

Figure S5. Model of the LPS biosynthetic pathways and receptors recognized by MLP phages. Schematic representation of O-antigen biosynthesis process by the Wzx/Wzy-dependent assembled pathway and LPS structure. Phages are represented by MLP1 (red), MLP2 (blue), and MLP3 (yellow). Mutations in genes (variants) identified by whole genome sequencing (WGS) of phage-resistant mutants are represented as boxes of the same color of the respective phage. Lambda head decoration protein D is depicted as a red circle in the extra and intracellular localization since its role in the MLP1 infection process is still unknown. Created with BioRender.com.

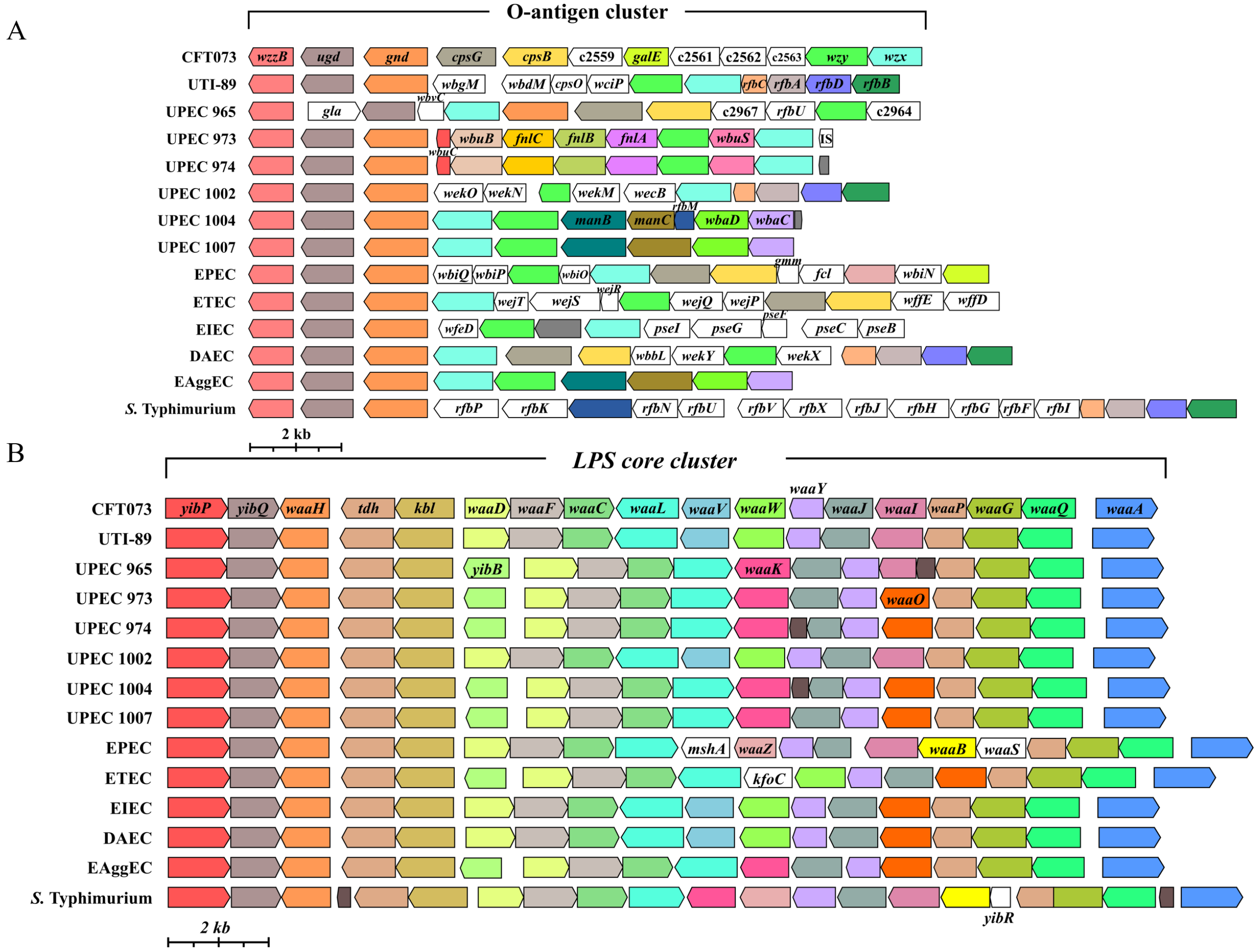

Figure S6. Comparison of genomic organization of LPS cluster between strains.

Gene content and cluster arrangement of O-Antigen (A) and LPS core clusters (B) for all strains used in this study.

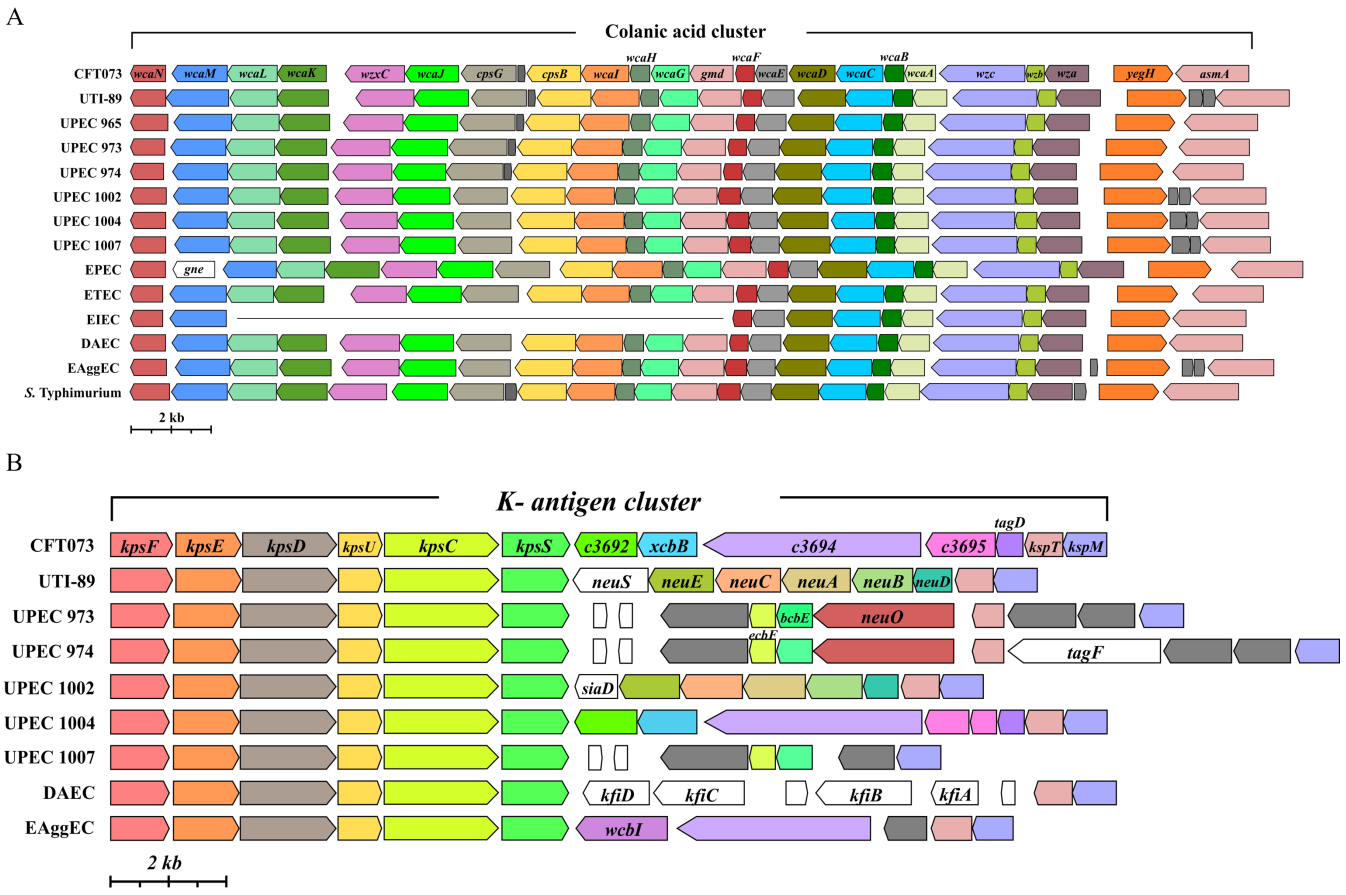

Figure S7. Comparison of genomic organization of capsule cluster between strains.

Genomic structure gene content and organization for colonic acid (A) and K-antigen clusters (B) for all strains used in this study.

Name / Strain #	Relevant genotype	Reference
CFT073 (ML1)	Wild type strain	Laboratory collection
Δ*waaL* (ML45)	Δ*waaL*::kan in the CFT073 background	This study
UTI-89 (ML209)	Wild type strain	Laboratory collection
UPEC 965 (ML56)	MDR clinical isolate from patient with UTI	This study
UPEC 973 (ML55)	MDR clinical isolate from patient with UTI	This study
UPEC 974 (ML57)	MDR clinical isolate from patient with UTI	This study
UPEC 1002 (ML60)	MDR clinical isolate from patient with UTI	This study
UPEC 1004 (ML59)	MDR clinical isolate from patient with UTI	This study
UPEC 1007 (ML58)	MDR clinical isolate from patient with UTI	This study
EPEC 2348/69 (ML47)	Intestinal pathogenic *E. coli* strain	Provided by Dr. Roberto Vidal
ETEC H10407 (ML48)	Intestinal pathogenic *E. coli* strain	Provided by Dr. Roberto Vidal
EIEC EI-34 (ML49)	Intestinal pathogenic *E. coli* strain	Provided by Dr. Roberto Vidal
EAggEC 034 (ML50)	Intestinal pathogenic *E. coli* strain	Provided by Dr. Roberto Vidal
DAEC F-1845 (ML51)	Intestinal pathogenic *E. coli* strain	Provided by Dr. Roberto Vidal
Enterobacter cloacae ATCC 23355 (ML143)	Wild type strain	Laboratory collection
Shigella flexneri 2457T (ML241)	Wild type strain	Laboratory collection
Salmonella Typhimurium 14028s (ML40)	Wild type strain	Laboratory collection
CFT073 E1M1G (ML348)	CFT073 resistant mutant to MLP1. WGS showed a variant in c1573:p.Val109Leu	This study
CFT073 E1M2H (ML345)	CFT073 resistant mutant to MLP2. WGS showed a variant in *rfaH*, c4789:p.Glu19*	This study
CFT073 E1M2G (ML352)	CFT073 resistant mutant to MLP2. WGS showed a variant in *rfaH,* c4789:p.Ser97*	This study
CFT073 E1M2B (ML351)	CFT073 resistant mutant to MLP2. WGS showed a variant in *rfaI,* c4453:p.Leu229*	This study
CFT073 E1M2A (ML350)	CFT073 Resistant mutant to MLP2. WGS showed a variant in *rfaG*, c4455:p.Ala219fs	This study
CFT073 E1M3H (ML354)	CFT073 Resistant mutant to MLP3. WGS showed a variant in c3693:p.Lys94fs	This study
CFT073 E1M3C (ML355)	CFT073 Resistant mutant to MLP3. WGS showed a variant in c2564:p.Asn404fs	This study
CFT073 E1M3E (ML356)	CFT073 Resistant mutant to MLP3. WGS showed a variant in c3693:p.Phe95fs	This study
CFT073 E1M3D (ML357)	CFT073 Resistant mutant to MLP3. WGS showed a variant in c2559:p.Val178fs	This study

Table S1. Strains used in this study.

Strain		Ampicillin	Ciprofloxacin	Nalidixic acid	Sulfamethoxazole	Azithromycin	Tetracycline
CFT073		-	-	-	-	-	-
UTI89		-	-	-	-	-	-
UPEC965	WGS-predicted phenotype	-	-	-	Resistant	-	Resistant
	Genetic background	-	-	-	sul2 (sul2_AY034138)	-	tet(A) (tet(A)_AF534183)
UPEC973	WGS-predicted phenotype	Resistant	-	-	Resistant	-	Resistant
	Genetic background	blaTEM-1B (blaTEM-1B_AY458016)	-	-	sul2 (sul2_HQ840942), sul1 (sul1_EU780013)	-	tet(A) (tet(A)_AF534183)
UPEC974	WGS-predicted phenotype	Resistant	-	-	Resistant	-	Resistant
	Genetic background	blaTEM-1B (blaTEM-1B_AY458016)	-	-	sul1 (sul1_U12338), sul2 (sul2_HQ840942)	-	tet(A) (tet(A)_AF534183)
UPEC1002	WGS-predicted phenotype	-	-	-	-	-	-
	Genetic background	-	-	-	-	-	-
UPEC1004	WGS-predicted phenotype	Resistant	-	-	Resistant	Resistant	-
	Genetic background	blaTEM-1B (blaTEM-1B_AY458016)	-	-	sul1 (sul1_EU780013), sul2 (sul2_HQ840942)	mph(A) (mph(A)_D16251)	-
UPEC1007	WGS-predicted phenotype	Resistant	Resistant	Resistant	Resistant	-	-
	Genetic background	blaTEM-1B (blaTEM-1B_AY458016)	gyrA (p.S83L)	gyrA (p.S83L)	sul2 (sul2_HQ840942), sul1 (sul1_EU780013)	-	-

Table S2. Antibiotic resistance genes present in the genomes of UPEC clinical isolates.
